# The role of head circumference and cerebral volumes to phenotype male adults with autism spectrum disorder

**DOI:** 10.1002/brb3.2460

**Published:** 2022-02-03

**Authors:** Niklaus Denier, Gerrit Steinberg, Ludger Tebartz van Elst, Tobias Bracht

**Affiliations:** ^1^ Translational Research Center University Hospital of Psychiatry and Psychotherapy University of Bern Bern Switzerland; ^2^ Department of Psychiatry and Psychotherapy Faculty of Medicine Medical Center‐University of Freiburg University of Freiburg Freiburg Germany

**Keywords:** autism spectrum disorder, biomarker, classification, head circumference, machine learning, phenotype

## Abstract

**Background:**

Autism spectrum disorder (ASD) has been repeatedly associated with enlargements of head circumference in children with ASD. However, it is unclear if these enlargements persist into adulthood. This is the first study to investigate head circumference in a large sample of adults with ASD.

**Methods:**

We apply a fully automated magnetic resonance imaging (MRI) based measurement approach to compute head circumference by combining 3D and 2D image processing. Head circumference was compared between male adults with ASD (*n* = 120) and healthy male controls (*n* = 136), from the Autism Brain Imaging Data Exchange (ABIDE) database. To explain which brain alterations drive our results, secondary analyses were performed for 10 additional morphological brain metrics.

**Results:**

ASD subjects showed an increase in head circumference (*p* = .0018). In addition, ASD patients had increased ventricular surface area (SA) (*p* = .0013). Intracranial volume, subarachnoidal cerebrospinal fluid (CSF) volume, and gray matter volume explained 50% of head circumference variance. Using a linear support vector machine, we gained an ASD classification accuracy of 73% (sensitivity 92%, specificity 68%) using head circumference and brain‐morphological metrics as input features. Head circumference, ventricular SA, ventricular CSF volume, and ventricular asymmetry index contributed to 85% of feature weighting relevant for classification.

**Conclusion:**

Our results suggest that head circumference increases in males with ASD persist into adulthood. Results may be driven by morphological alterations of ventricular CSF. The presented approach for an automated head circumference measurement allows for the retrospective investigation of large MRI datasets in neuropsychiatric disorders.

## Graphical abstract

We investigated by a novel and fully automated MRI‐based measurement approach head circumference and additional brain morphological metrics in a large publicly available dataset of an adult male with autism spectrum disorder (ASD) and healthy controls. We found increased head circumference in ASD, mostly explained by alterations in cerebrospinal fluid volume.

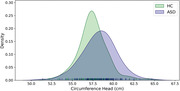

## 1 INTRODUCTION

Autism spectrum disorder (ASD) is a neurodevelopmental disorder characterized by severe and persistent deficits regarding social communication and interactions and repetitive behavioral patterns or narrowed interests (APA, 2013). The detection of infants with high‐risk profiles for the development of ASD is of clinical relevance because this allows for early therapeutic interventions, which have been shown to be beneficial for the clinical course (Rogers et al., 2014). Clinical markers for high‐risk infants in ASD include poor eye contact, impaired affective reciprocity, and impairments of joint attention skills (Barbaro & Dissanayake, 2013). However, ASD symptoms overlap with other developmental disorders, which hamper an early diagnosis (Sacco et al., 2015). Furthermore, ASD patients without accompanying intellectual impairments may compensate or mask initial symptoms in early childhood (van Elst et al., 2013).

Biomarkers may complement clinical assessments and contribute to a neurobiological informed risk assessment to develop ASD (Goldani et al., 2014). One of the most frequently reported phenotypes in ASD is an enlarged head circumference (Sacco et al., 2015). The majority of studies suggest that head circumference in ASD is average at birth (Courchesne et al., 2001; Sacco et al., 2015). Increases in head growth have been reported repeatedly within the first 4 years of life (Mraz et al., 2007; Sacco et al., 2007; Schumann et al., 2010). While there is an extensive body of literature on head circumference in young children (Morhardt et al., 2014; Sacco et al., 2015), there are uncertainties about head circumference alterations in adolescents and adults with ASD (Sacco et al., 2015). Some studies reported enlarged head circumference in samples including adults with ASD (Aylward et al., 2002; Lainhart et al., 2006; Sacco et al., 2015; Stevenson et al., 1997). However, conclusions on adults with ASD are limited because those studies also incorporated children and adolescents and had small sample sizes.

Consequently, the question arises if increases in head circumference persist even further until adulthood. Therefore, this study aimed at investigating head circumference in a large group of adults with ASD. To increase homogeneity, only adult males were included. In contrast to previous studies measuring head circumference by hand, we assessed head circumference using a fully automated approach based on magnetic resonance imaging (MRI) data.

Increases in head circumference in ASD are likely to be reflected by increases of cerebral growth (Sacco et al., 2015). Cerebral overgrowth in ASD has been reported in particular in the first years of life (Courchesne et al., 2003; Hazlett et al., 2017). In a recent study, it was hypothesized that negative findings in older patients with ASD may be due to exclusion of more severely ill ASD patients with megalencephaly (Lee et al., 2020). Results of this previous longitudinal study point to persisting cerebral overgrowth in boys at least until late childhood (Lee et al., 2020); a finding that is in line with another longitudinal study that did not find a volumetric regression of brain growth in children with ASD (Libero et al., 2016). In addition, some studies reported brain overgrowth in adults with ASD (Piven et al., 1995; Tsatsanis et al., 2003). To investigate if putative head circumference increase in adult ASD is associated with alterations of cerebral volume and morphology, we also computed intracranial volume (ICV), total brain volume, grey matter (GM) volume, white matter (WM) volume (WM), total cerebrospinal fluid (CSF) volume, subarachnoidal CSF volume, ventricular CSF volume, ventricular surface area (SA), ventricular asymmetry index (AI), and total CSF AI.

We hypothesized increased head circumference in male adults with ASD. We assumed that those increases in head circumference underlie alterations of cerebral volumes of brain compartments. Additionally, we investigated the putative role of head circumference as a phenotype biomarker of ASD. We performed a machine learning approach to classify ASD patients based on head circumference and above‐mentioned complementary brain‐morphological metrics and investigate their role in the successful stratification of male adults with ASD.

## 2 METHODS

### 2.1 Study sample

We used 3‐Tesla T1‐weighted structural MRIs with a voxel size of 1 × 1 × 1 mm from the Autism Brain Imaging Data Exchange I (ABIDE I) sample (https://fcon_1000.projects.nitrc.org/indi/abide/abide_I.html). The ABIDE I sample consists of 1112 subjects, including 539 subjects with ASD and 573 typically developing healthy controls (HC). For our analyses, we chose an age threshold of ≥20 years to ensure completion of brain development (Arain et al., 2013). To increase the homogeneity of our analyses, we included only adult males (Ferri et al., 2018). Both right‐handers, left‐handers, and ambidextrous participants were included (assessment not specified) (Di Martino et al., 2014). This resulted in an ABIDE I subsample comprised of 120 adult males with ASD and 136 healthy male controls (HC). Only a small sample of adult females could be found in the dataset. Analyses of head circumference of 33 adult females (14 ASD, 19 HC) are shown in Figure S4.

### 2.2 Image pre‐processing

MRI volumes were pre‐processed using MATLAB R2021a, statistical parametric mapping (SPM 12), and MATLAB's Image Processing Toolbox. First, using SPM 12 segmentation and normalization procedures, native space T1‐weighted structural images were segmented into five different tissue types (GM, WM, CSF, bone structures, soft tissue of head and neck) for further processing of head metrics.

### 2.3 Computation of head circumference

**FIGURE 1:**
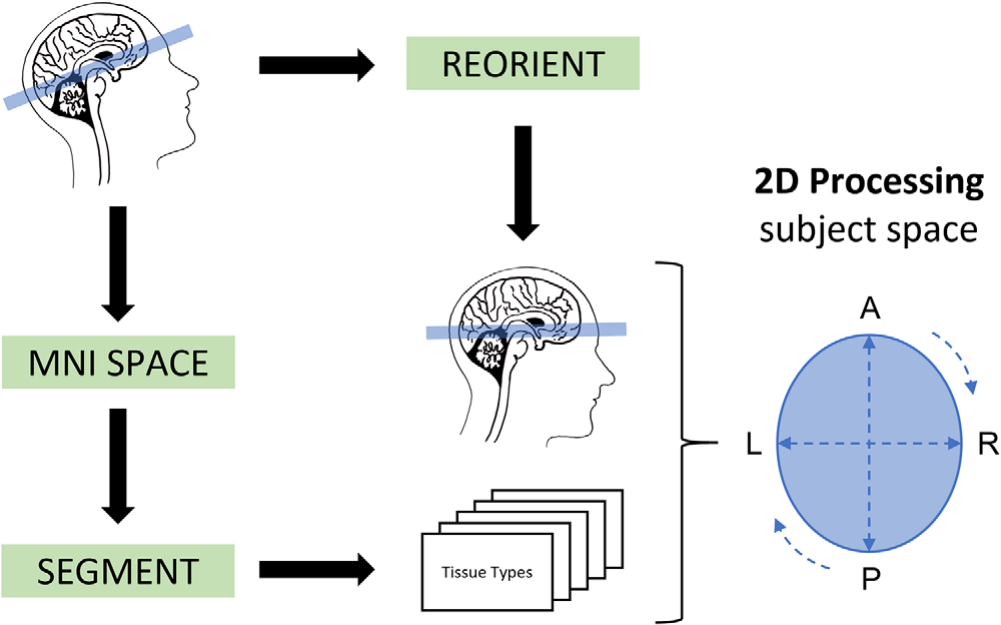
Fully automated MRI‐based pipeline of head circumference measurement using 3D and 2D image processing

T1‐weighted structural images and their corresponding tissue types were reoriented according to the MNI space's alignment using rigid‐body transformation (rotation and translation) to obtain a standardized head alignment. The standardized plane for measuring the head circumference, corresponding to MNI z = 0, included the middle of the forehead and the most prominent portion of the occipital bone (occiput). For measurement of head circumference, we combined all five tissue types ($\sum_{i\;=\;1}^{5}\mathrm{tissue}\;\mathrm{type}\;i)$, applied a smoothing kernel of 4 × 4 × 4 mm^3^, and binarized it with a threshold of t>0.5. Using the *regionprops* function of the Image Processing Toolbox, we computed the perimeter of the plane (head circumference) and its primary length and width (see Figure 1). To investigate if differences in head circumference are driven by alterations of head shape, we also calculated the cephalic index (CI): $\mathrm{CI}\;=\frac{\mathrm{width}}{\mathrm{length}}\;\times \;100$. The human head can be classified in the following shape types: dolichocephalic (CI: 70–75), mesocephalic (CI: 75–80), brachycephalic (CI: 80–85), and hyperbrachycephalic (CI: 85–90) (Franco et al., 2013). In most humans, there is a mesocephalic shape (Ahluwalia et al., 2020). (See Figure S1.) Additional to head circumference, we computed the area of head, ICV, and brain (in cm^2^) in the corresponding 2D plane. (See Figure S2A.) We tested the reliability of our automated head circumference measurement using a small internal dataset with healthy subjects (*n* = 6) and gained a Cronbach's α of 0.86.

### 2.4 Computation of intracranial metrics

All ICVs were calculated by the sum of the probability values (0…1) of all voxels (resolution 1 mm^3^) of the corresponding individual tissue probability map (GM, WM, CSF). ICV and brain volume were computed by combining individual probability maps of GM, WM, and CSF tissue types ($\mathrm{ICV}\;=\sum_{i=1}^{3}\mathrm{tissue}\;\mathrm{type}\;i\;$, $\mathrm{brain}\;=\;\sum_{i\;=\;1}^{2}\mathrm{tissue}\;\mathrm{type}\;i$) and sum of the probability values of all voxels (resolution 1 mm^3^). Ventricular and subarachnoidal CSF volumes were computed using the ventricular automatic lateral ventricle delineation (ALVIN) mask (Kempton et al., 2011) and by calculating its intersection and difference with the CSF probability maps:ventricles=totalCSF∩mask, subarachnoidalCSF=totalCSF−ventricles. To assess hemispherical CSF asymmetry, we computed AI in ventricular and total CSF: $\mathrm{AI}\;=\;\frac{\mathrm{right}-\mathrm{left}}{\mathrm{right}+\mathrm{left}}$ (Palmer & Strobeck, 1986) and absolute (abs(left−right)) differences in hemispherical ventricular and total CSF to show absolute differences. (See Figure S3.) Additionally, we used a marching cubes algorithm implemented in MATLAB (Hammer, 2021) to compute a triangulated mesh of the isosurface of the ventricle, which we used for measuring ventricular SA in cm^2^.

### 2.5 Statistical analyses

Statistical analyses were performed using Statistical Package for Social Sciences SPSS 26.0 (SPSS, Inc., Chicago, Illinois). Independent t‐tests were used to calculate group differences between ASD and HC regarding age and full‐scale IQ (assessment not specified), (Di Martino et al., 2014). Handedness was compared between ASD and HC using a $\chi^{2}$ test. For group comparisons of head circumference between ASD and HC, we used an analysis of covariance (ANCOVA) with the independent variable group (ASD, HC), and the dependent variable head circumference controlling for age. The effect size was computed using Cohen's d. Independent *t*‐tests were used to calculate group differences between the CI and area measurements in the 2D plane of head, ICV, and brain.

To explore group differences of our secondary morphological outcome measures, we used a MANCOVA controlling for age with the independent variable group (ASD, HC) and the dependent variables ICV, total brain volume, GM volume, WM volume, total CSF volume, subarachnoidal CSF volume, ventricular CSF volume, ventricular SA, ventricular AI, and total CSF AI. Additionally, we tested absolute hemispherical differences of ventricular CSF volume and total CSF volume with ANCOVA's controlling for age and corresponding ventricular or total CSF volume.

To investigate associations between head circumference and morphological brain metrics a stepwise linear regression across all participants was used. The dependent variable was head circumference. The independent variables were the following brain morphological variables: ICV, total brain volume, GM volume, WM volume, total CSF volume, subarachnoidal CSF volume, ventricular CSF volume, ventricular SA, ventricular AI, and total CSF AI.

### 2.6 Support vector machine

A support vector machine (SVM) with a linear kernel implemented in Python's *sklearn* package was used for training and testing the classification of ASD and HC. For training, we used standard parameters including a regularization parameter C = 1, a squared hinge loss function, and an l2 norm penalization. The ASD and HC groups were split and stratified in a training (80%, *n* = 204) and a test (20%, *n* = 52) sample. Head circumference, ICV, total brain volume, GM volume, WM volume, CSF volume, subarachnoidal CSF volume, ventricular CSF volume, ventricular SA, ventricular AI, and total CSF AI were all rescaled (0 to 1) and used as input features to train an 11‐dimensional separation hyperplane. Using the independent test sample, we calculated classification accuracy ($\frac{\mathrm{TP}\;+\;\mathrm{TN}}{\mathrm{TP}\;+\;\mathrm{TN}\;+\;\mathrm{FP}\;+\;\mathrm{FN}}$), sensitivity ($\frac{\mathrm{TP}}{\mathrm{TP}+\mathrm{FN}}$), and specificity ($\frac{\mathrm{TN}}{\mathrm{TN}+\mathrm{FP}}$), of the classification model, where TP is true positive, FP is false positive, TN is true negative, and FN is false negative. The 11 different feature metrics were additionally investigated by calculating their percentual weight contribution for classification.

## 3 RESULTS

### 3.1 Study population

Groups did not differ significantly regarding age (HC: 27.6 ± 6.9 years, ASD: 28.6 ± 8.7 years, *t*_254_ = 1.011, *p* = .313). Subjects including an assessment of full‐scale IQ (HC: *n* = 108, ASD: *n* = 108) did not differ significantly between groups (HC: 111 ± 15, ASD: 109 ± 15, *t*_214_ = −1.005, *p* = .316, missing: *n* = 40). Groups did not differ significantly in handedness ($\chi^{2}$ = 2.131, df = 3, *p* = .546, missing: *n* = 113).

### 3.2 Group comparisons

**FIGURE 2:**
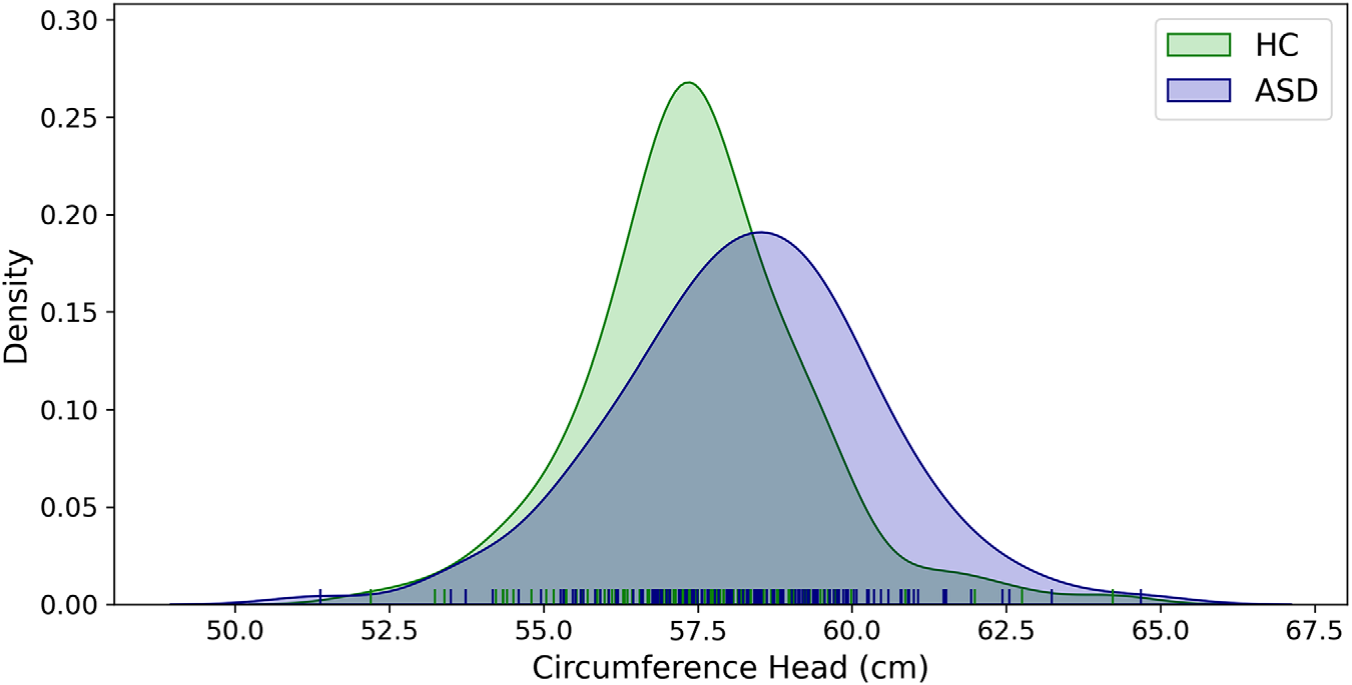
Group distribution of head circumference in HC and ASD

The ANCOVA revealed a significant main effect of group on head circumference controlling for age (F_1, 256_ = 9.995, *p* = .0018, ASD: 58.3 ± 2.1 cm, HC: 57.4 ± 1.8 cm, *d* = 0.41). (See Figure 2.) Groups did not differ regarding CI (HC: 77.9 ± 3.3 years, ASD: 77.5 ± 3.3, *t*_254_ = 0.882, *p* = .378) suggesting that head circumference increases in ASD are not influenced by the shape of the head. However, we found an increased area of the head in the 2D measurement plane in ASD patients (HC: 263 ± 15 cm^2^, ASD: 256 ± 13 cm^2^, *t*_254_ = 3.772, *p* = .0002. *d* = 0.47), but no significant differences in ICV (HC: 183 ± 14 cm^2^, ASD: 183 ± 17 cm^2^, *t*_254_ = 0.112, *p* = .911) and brain volume (HC: 171 ± 14 cm^2^, ASD: 170 ± 19 cm^2^, *t*_254_ = 0.413, *p* = .680). See Figure S2B.

The MANCOVA revealed a significant main effect of group (F_6, 248_ = 2.742, *p* = .013). Post hoc tests revealed a significantly higher ventricular SA (ASD 35 ± 14 ml, HC 31 ± 8 ml, *p* = .0013) and a significantly higher ventricular CSF volume (ASD 35 ± 14 ml, HC 31 ± 8 ml, *p* = .0013). No significant group differences were found in ICV, total brain volume, WM volume, GM volume, total CSF volume, subarachnoidal CSF volume, total CSF AI, and ventricular CSF AI. (See Table 1.) We found no significant absolute ventricular CSF volume differences in ASD subjects (HC: 1.25 ± 1.2 ml, ASD: 1.72 ± 1.9 ml, F_1,256 _= 1.068, *p* = .303) and absolute total CSF volume differences (HC: 6.5 ± 4.4 ml, ASD: 6.8 ± 4.9 ml, F_1,256 _= 0.100, *p* = .751).

**TABLE 1:** Group comparison of head circumference and brain‐morphological metrics between HC and ASD

	**HC (*n* = 120)**	**ASD (*n* = 136)**	***p*‐Values**
Head circumference (cm)	57.4 (1.8)	58.3 (2.1)	0.0018**
ICV (ml)	1579 (119)	1600 (152)	0.219
Total brain volume (ml)	1286 (102)	1300 (133)	0.217
GM volume (ml)	794 (71)	806 (91)	0.070
WM volume (ml)	491 (48)	493 (58)	0.859
Total CSF volume (ml)	294 (72)	301 (94)	0.070
Subarachnoidal CSF volume (ml)	276 (69)	279 (90)	0.748
Ventricular CSF volume (ml)	18 (6)	21 (10)	0.003**
Ventricular SA (cm^2^)	104 (17)	113 (24)	0.001**
Ventricular AI	0.029 (0.020)	−0.033 (0.024)	0.075
Total CSF AI	0.012 (0.0049)	0.013 (0.0050)	0.8877

** *p* < .01.

**FIGURE 3:**
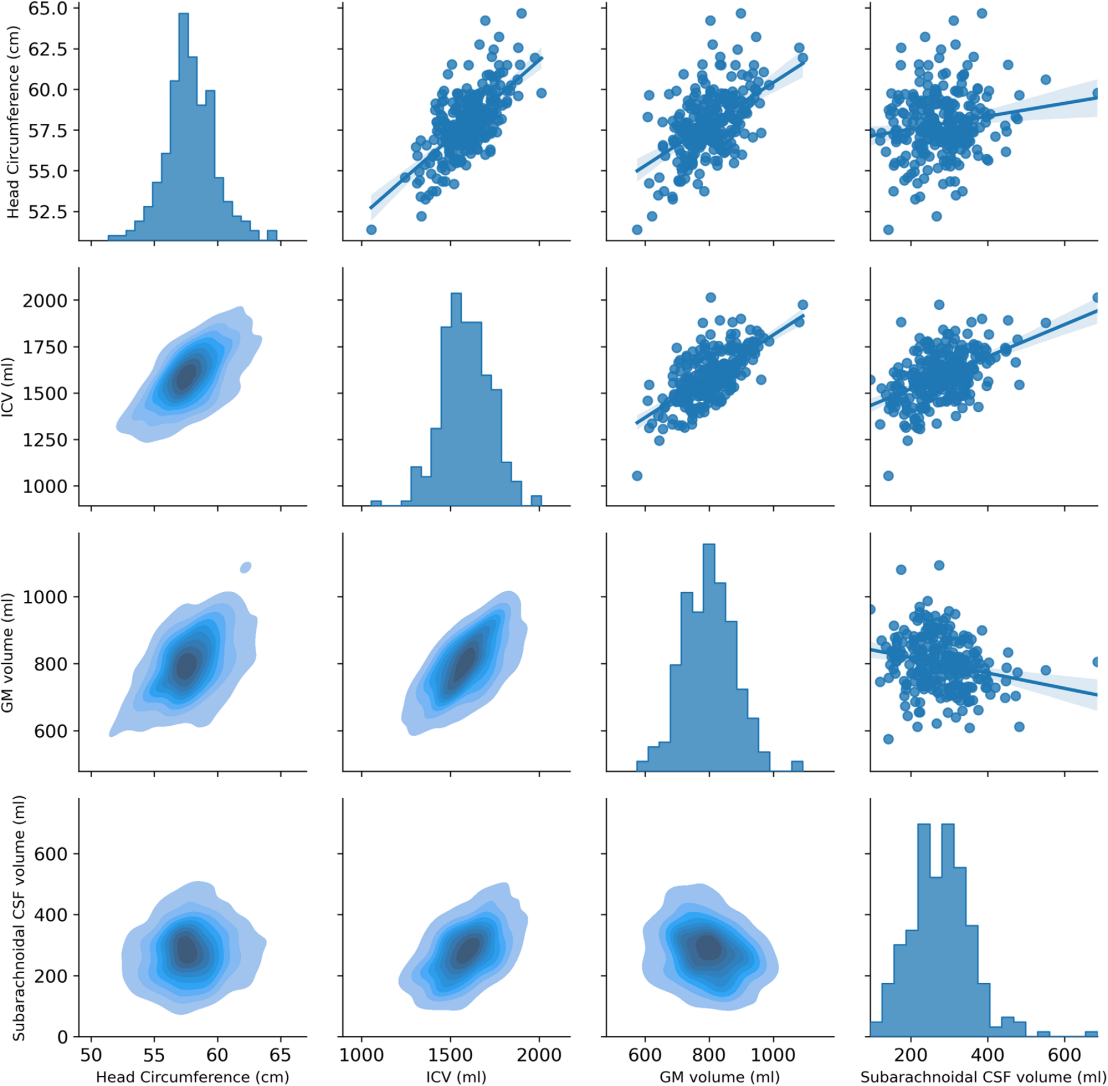
Pair plot correlation of head circumference, subarachnoidal CSF volume, ventricular CSF volume, and ventricular SA

Using a stepwise linear regression analysis, ICV (R^2^ = 0.427), subarachnoidal CSF volume (cumulated R^2^ = 0.465), and GM volume (cumulated R^2^ = 0.476) were included in the final model (F_4, 255_ = 78.154, *p* < .001) explaining 50 % of head circumference variance. For visualization purposes, we display correlations between head circumference and brain morphological measures included in the stepwise linear regression. (See Figure 3.)

### 3.3 Support vector machine

**FIGURE 4:**
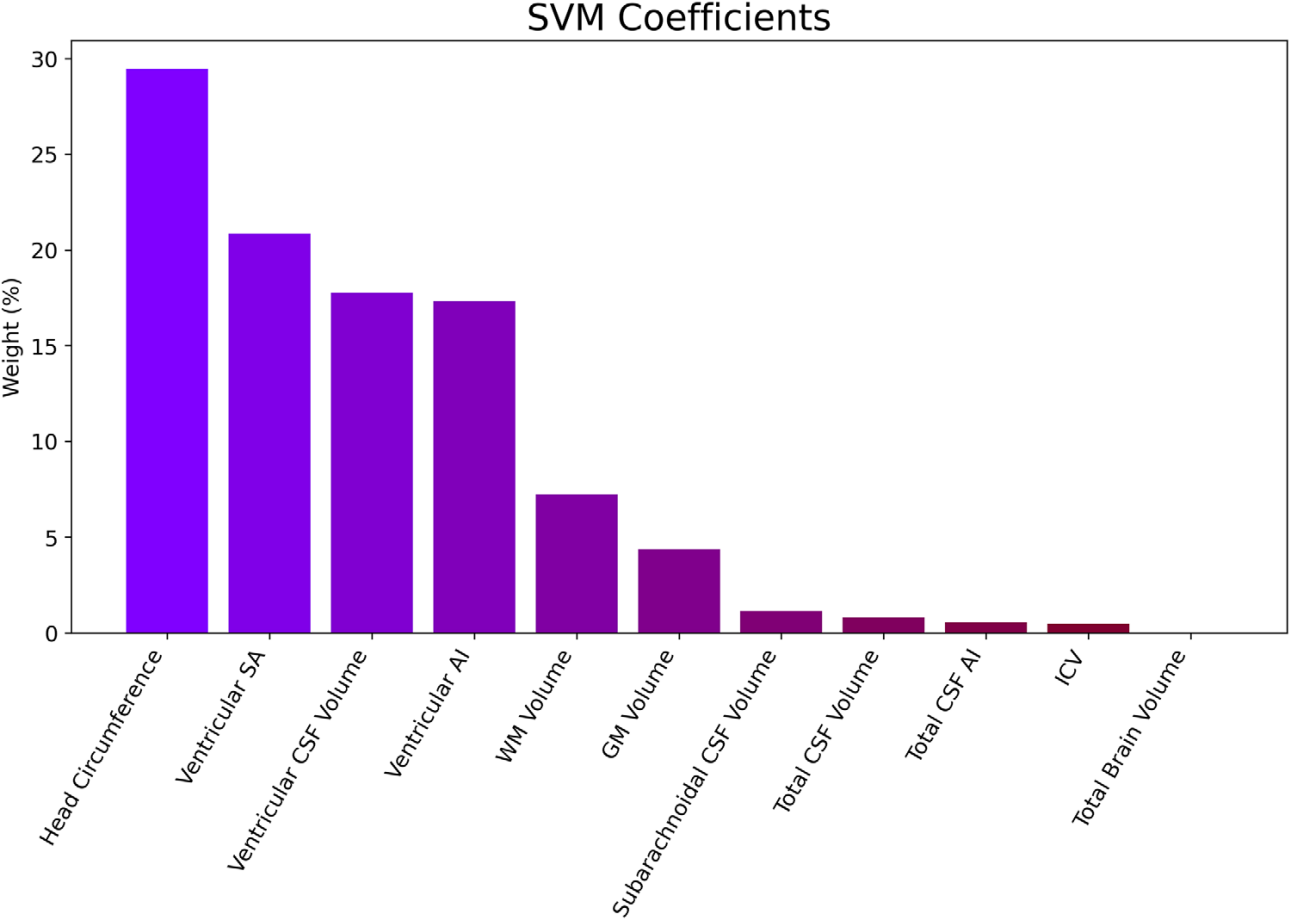
Percentual weight of head circumference and brain‐morphological features relevant for SVM classification

In the independent test dataset, the linear SVM classifier reached a classification accuracy of 73% with a corresponding sensitivity of 92% and specificity of 68%. The feature weights of head circumference, ventricular SA, ventricular CSF volume, and ventricular AI contributed most to classification (85%). (See Figure 4.)

## 4 DISCUSSION

This is the largest study investigating head circumference in adult males with ASD and the first study to use an MRI‐based fully automated measurement approach. We found an increase in head circumference in our male ASD sample. In addition, there were significantly higher ventricular SA and ventricular CSF volumes in ASD subjects. ICV, subarachnoidal CSF volume, and GM volume explained 50% of head circumference variance. Based on a machine learning classification with a linear SVM, we gained a classification accuracy of 73% (sensitivity 92%, specificity 87%), whereas head circumference and ventricular metrics (volume, SA, AI) contributed to 85% of feature weighting relevant for classification.

We identified increases in head circumference in adults with ASD. While a series of studies reported head circumference increases in infants with ASD (Sacco et al., 2015), studies including adults with ASD are rare. So far, all those studies used manual measurements of head circumference and identified increases in head circumference in small samples of adults with ASD (Aylward et al., 2002; Lainhart et al., 2006; Sacco et al., 2015; Stevenson et al., 1997), which is in line with our finding. However, a direct comparison of these previous studies with our findings is difficult because all these studies included infants and adolescents in their ASD sample, which may have driven significant results. In contrast, we investigated a homogenous adult sample enabling clear conclusions on male adults with ASD. Our study is by far the largest study to investigate head circumference in adult ASD. Given the moderate effect size of our finding (*d* = 0.4), it is possible that previous studies were underpowered. Based on power analysis for mean comparisons between two groups (using an effect size of *d* = 0.4, α‐error of *p* = .05 (two‐tailed), and a power (1‐β) of 0.8), a sample size of 100 participants per group would be required to detect such a difference (Faul et al., 2007). Thus, one may speculate whether there is a publication bias with unpublished false‐negative results.

Secondary analysis of cerebral volumes and morphology revealed higher ventricular SA and ventricular CSF volumes in ASD in contrast to HC. This suggests that our finding of increased head circumference may also underlie volumetric and morphological alterations of CSF and the ventricular system. In line with this observation, previous studies found increases of CSF volume in infants who later develop ASD (Shen et al., 2017; Shen et al., 2018), as well as in adolescents (McAlonan et al., 2005). Our study complements findings of a previous study of the ABIDE data set reporting CSF increases in a mixed‐gender sample aged between 16 and 22 years but not in adults older than 22 years of age (Riddle et al., 2017). Thus, it is possible, that CSF increases in adults with ASD are specific for males. Our analyses further extend previous publications because we differentiate between subarachnoidal and ventricular CSF volume in adults. In our study, only ventricular CSF volume but not subarachnoidal CSF volume differed between groups. This methodological refinement may also explain diverging results regarding the CSF comparisons performed by (Riddle et al., 2017).

We did not identify significant differences in ventricular and total CSF AI. Measurements of hemispherical asymmetry in ASD are sparse and heterogeneous. One study used a large dataset of the ENIGMA consortium and revealed reduced cortical thickness asymmetry in medial frontal, orbitofrontal, cingulate, and inferior temporal areas in infants, adolescents, and adults with ASD (Bartholomeusz et al., 2002). Another study found increased lateral ventricle volume asymmetry in ASD, including children, adolescents, and young adults (Richards et al., 2020). Further research is warranted to elucidate the presence and the role of brain asymmetry in ASD (Floris et al., 2020).

Across all participants, ICV explained by far most of the variance of head circumference (*R* ^2^ = 0.427), which is in line with previous findings (Hshieh et al., 2016). Our additional 2D measurements did not reveal significant area differences in ICV in contrast to the head area. However, due to missing information about 3D head shape due to the anonymization process, we cannot conclude higher impact of head shape (including bones and soft tissues) over ICV. Previous reports suggest that the strong association between head circumference and brain volume in toddlers and infants declines during adolescence and adulthood (Bartholomeusz et al., 2002). These results indirectly support our findings of non‐significant differences in ICV and brain volume between ASD and HC.

Head circumference is an easy to obtain and reproducible measure. Our results suggest head circumference increases in ASD and may thus serve as a phenotype biomarker in ASD. Machine learning combining and testing different measurement features may help to elucidate such phenotypic markers' suitability in adult ASD. We used a linear SVM to investigate classification accuracy using eleven MRI‐derived morphological metrics. Out of these measures, head circumference provided the most significant contribution for classification. This underlines the putative role of head circumference as a phenotypic marker in adult ASD. However, due to the considerable overlap of head circumference between ASD and HC, this sole measure provides insufficient information for a clinical application. Head circumference, ventricular SA, ventricular volume, and ventricular AI accounted for 85% of classification accuracy. This points to a crucial role of ventricular morphological properties, which may underlie head circumference increases in adult ASD.

Heterogeneity in the neuroanatomical phenotype of ASD across individuals makes it challenging to identify reliable phenotypic markers (Ecker, 2017). Nevertheless, the head circumference measurement provides an easy‐to‐obtain measure and may represent a novel phenotype for ASD stratification in adults. In conjunction with other phenotypic markers, including genetic information (Autism Genome Project et al., 2007; Vorstman et al., 2017), CSF liquor measurements (Runge et al., 2020), behavioral assessments (Yaneva et al., 2020), and novel MRI metrics such as cortical gyrification or folding patterns (Kohli et al., 2019; Ni et al., 2020), classification accuracy may likely be further improved. Thus, head circumference as a geometric measurement may be of interest for multimodal classification and prediction approaches.

In this study, we apply an MRI‐based, fully automated measurement of the human head circumference. An advantage of this approach is that there are no user‐dependent inaccuracies compared to manual measurements (Bushby et al., 1992; Nellhaus, 1968). Previous studies also developed automated head circumferences measurements in fetal ultrasound imaging (Kim et al., 2019; Li et al., 2018; Zhang et al., 2017), but this is the first study applying an automatic measurement of head circumference in adults based on structural MRI. Our approach is applicable in a time‐efficient way and may allow for a retrospective investigation of this phenotype's role in large MRI datasets. Furthermore, additional parameters such as the CI could be derived easily.

Our study has some limitations. First, due to the lack of respective data, we cannot assess the influence of variables such as height, body mass index (BMI), or average parental head circumference on derived measurements. Under normal clinical circumstances, these variables are not strongly correlated with head circumference in infants (Scheffler et al., 2017). However, there may be a subtle influence in adults (Mansur et al., 2014). Second, it is not possible to generalize our results to women with ASD. However, due to nosological and diagnostic challenges between male and female ASD (Cauvet et al., 2020; Lai et al., 2015), we decided to investigate a homogeneous subgroup of male adults with ASD. Third, head circumference increases in ASD may be more pronounced in low‐functioning ASD (Gillberg & de Souza, 2002). Our study consisted of a sample with an IQ ranging from 65 to 148 (mean IQ = 110). Notably, group differences of head circumference between ASD and HC remain significant if only participants with IQ ≥ 100 are included (ANCOVA: F_2, 168 _= 9.390, *p* = .0025). Thus, our results also remain if only high‐functioning ASD patients are included. Fourth, the ABIDE I data set is composed of data from multiple study sites. Therefore, it was inevitable that MRI‐data specificities and assessment protocols of participants (e.g., IQ tests) were performed differently. However, such variables should have diminished the probability of detecting differences since they should increase noise. On the other hand, cumulating data allows for the investigation of large data sets, thereby substantially increasing statistical power. Fifth, we apply an automated approach to measure head circumference. However, due to the nature of this publicly available data set, it was impossible to compare our measure with manually derived measures of head circumference.

In conclusion, our study suggests the suitability of head circumference in conjunction with brain morphological measures to phenotype male adults with ASD. Increases in head circumference in ASD may stem from volumetric and morphological CSF alterations. We applied an automated and user‐independent head measurement approach which is applicable in a time‐efficient way. This paves the way for a retrospective investigation of this phenotype in large MRI datasets in ASD and in other neurodevelopmental disorders.

## CONFLICT OF INTEREST

The authors report no biomedical financial interests or potential conflicts of interest.

### PEER REVIEW

The peer review history for this article is available at https://publons.com/publon/10.1002/brb3.2460

## Supporting information

Supporting information

## Data Availability

The authors confirm that the data supporting the findings of this study are available within the article and its supplementary materials.
