# Supplementary material for: The role of head circumference and cerebral volumes to phenotype male adults with autism spectrum disorder
Source: Brain Behav. 2022 Feb 3;12(3):e2460. doi: 10.1002/brb3.2460 (PMC8933748; doi:10.1002/brb3.2460)
Supplement: Supplementary file 1 — Supporting information [file BRB3-12-e2460-s001.docx]

Supplementary Figures

**Supplementary Figure 1.** Distribution of head shape types in male HC and ASD


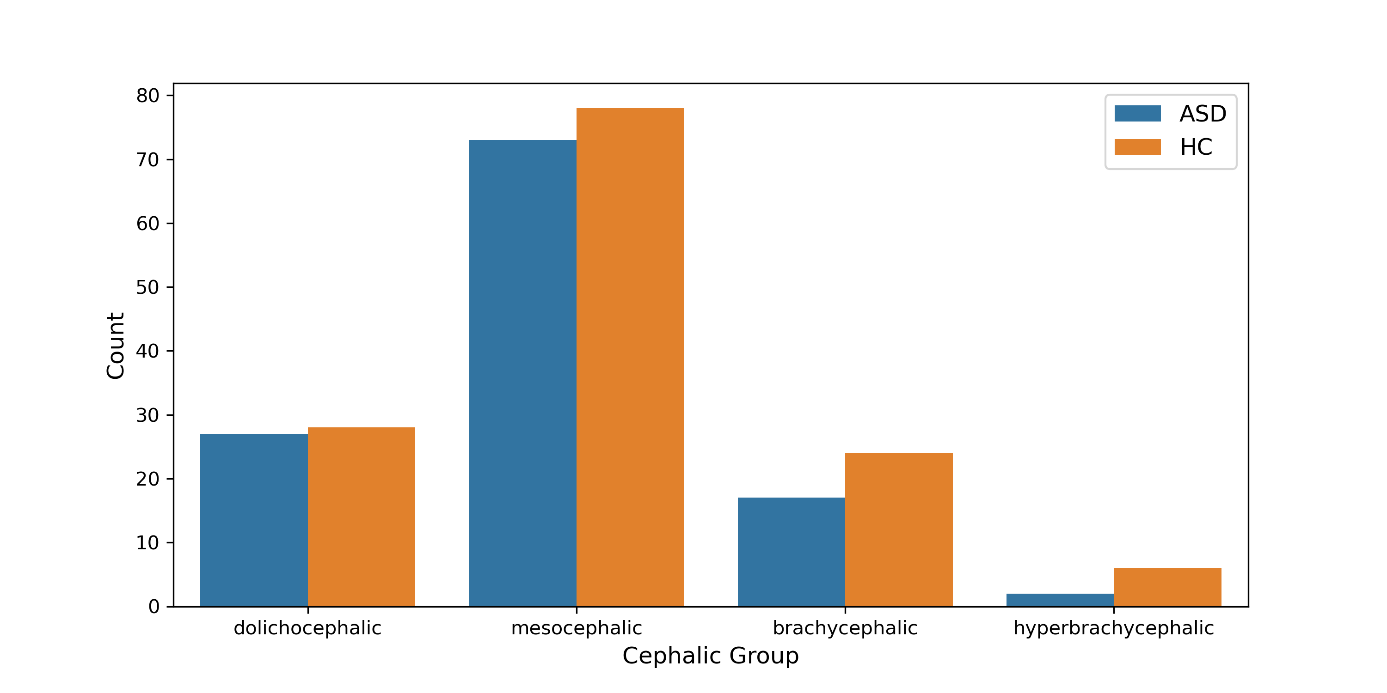


**Supplementary Figure 2.** Area measurements of head, ICV and brain.


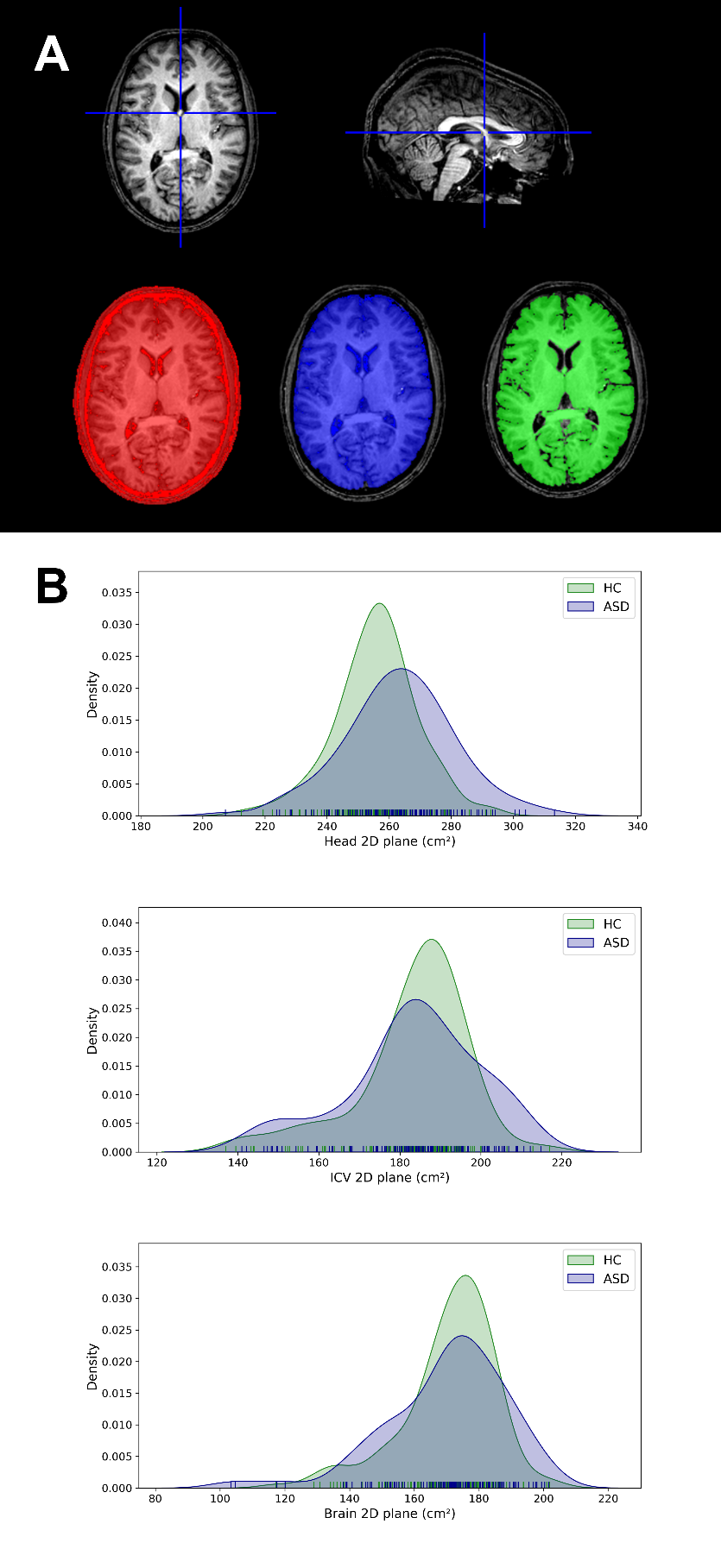


**A:** Plane for head circumference measurement with area of head (red), ICV (blue) and brain (green). **B:** Group distribution of head, ICV and Brain in HC and ASD.

**Supplementary Figure 3.** Pre-processing of MRI metrics.


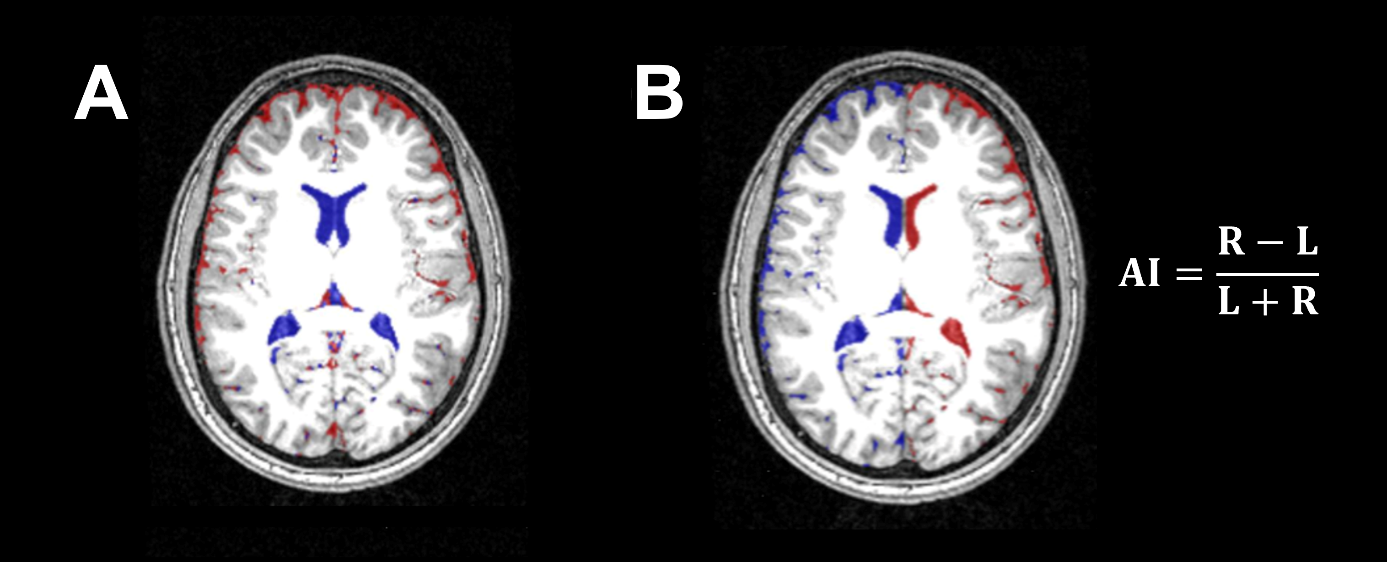


**A:** Segmentation of ventricle CSF (blue) and subarachnoidal CSF (red). **B:** Computation of AI between right (red) and left (blue) hemisphere.

**Supplementary Figure 4.** Group differences in head circumference and head shape in females.


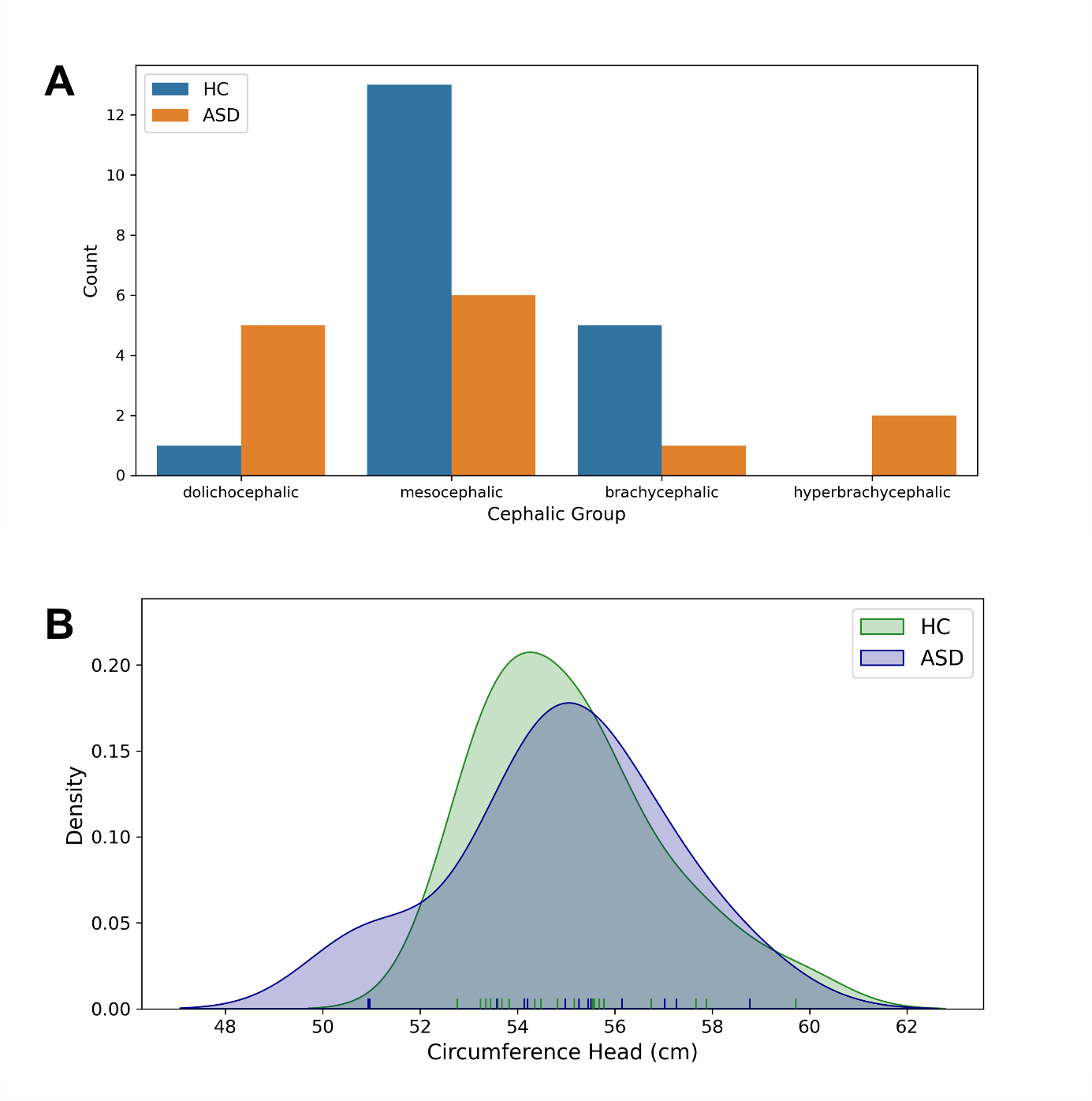


**A:** No significant group difference in head shape and cephalic index (p = 0.995). **B:** No significant difference in head circumference controlling for age (HC: 55.13 ± 1.79 cm, ASD: 54.89 ± 2.11 cm, F_1,32_ = 0.158, p = 0.694).
